# Establishment of an in vitro human blood-brain barrier model for antiviral screening against neurotropic viruses

**DOI:** 10.1186/s12987-026-00860-z

**Published:** 2026-09-24

**Authors:** Marisol Zuniga, Alexander Herrmann, Matteo Rizzato, Michal Himl, Adriana Fontes, Hans Zischka, Romano Silvestri, Andrea Brancale, Joachim J. Bugert, Ruth Brack-Werner

**Affiliations:** 1 Institute of Virology, Helmholtz Center Munich, German Research Center for Environmental Health, Ingolstädter Lanstraße 1, 85764 Neuherberg, Germany; 2 Institute of Molecular Toxicology and Pharmacology, German Research Center for Environmental Health, Helmholtz Center Munich, Ingolstädter Lanstraße 1, 85764 Neuherberg, Germany; 3 Institute of Toxicology and Environmental Hygiene, School of Medicine and Health, Technical University of Munich (TUM), Biederstenerstraße 29, 80802 Munich, Germany; 4 Department of Drug Chemistry and Technologies, Sapienza University of Rome, Piazzale Aldo Moro 5, Rome, 00185 Italy; 5 Department of Organic Chemistry, University of Chemistry and Technology Prague, Technická 5, Prague, 16628 Czech Republic; 6 Bundeswehr Institute of Microbiology, Neuherbergstraße 11, 80937 Munich, Germany; 7 Department of Biology ll, Ludwig-Maximilians-Universität (LMU), Großhaderner straße 2-4, 82152 Planegg-Martinsried, Germany

**Keywords:** Blood-brain barrier (BBB), Multicellular in vitro model, Antivirals, Neurotropic viruses, Tick-borne encephalitis virus (TBEV)

## Abstract

**Background:**

Neurotropic viruses infect the central nervous system (CNS) resulting in severe neurological and long-term complications, including permanent cognitive impairments. However, for many of these neurotropic viruses, like tick-borne encephalitis virus (TBEV), there are no clinically approved antiviral drugs. The blood-brain barrier (BBB) is a key entry point for neurotropic viruses, as well as a barrier for drug delivery to the brain. To aid in the identification of BBB-penetrating antivirals we established a BBB-permeable antiviral screening assay (BASA).

**Methods:**

BASA combines antiviral assays with an in vitro BBB transwell model consisting of primary human brain microvascular endothelial cells (HMBEC) and pericytes (HBMVP). The combination of our in vitro BBB transwell model with astrocyte-enriched cell cultures provided a tri-culture model to investigate TBEV infection at the BBB.

**Results:**

The suitability of BASA for identifying BBB-permeable antivirals was validated by testing clinically approved anti-human immunodeficiency virus (HIV) drugs with known CNS-penetrating properties. Infection experiments reveal that TBEV crosses the intact BBB to infect astrocytic target cells. Through the application of BASA, we identify a novel BBB-permeable antiviral against TBEV (compound 3d).

**Conclusions:**

We establish a modular assay system for the early evaluation of CNS penetration potential of novel antiviral compounds. The development of tools like ours can help promote early-stage preclinical optimization of dedicated therapeutics against neurotropic viruses, while reducing the need for animal experiments.

**Graphical Abstract:**

**Figure:**
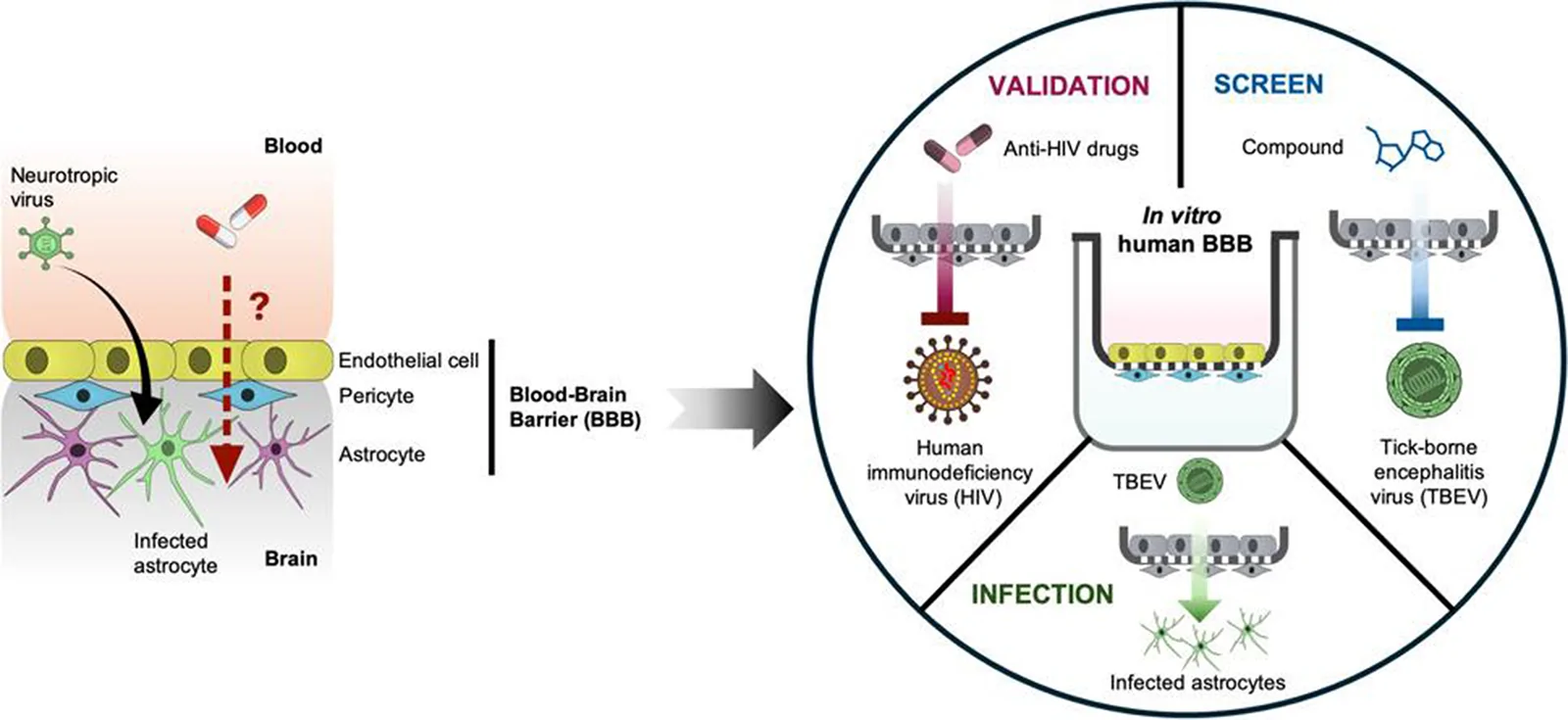

**Supplementary Information:**

The online version contains supplementary material available at https://doi.org/10.1186/s12987-026-00860-z.

## Background

The COVID-19 pandemic revealed not only the success of mRNA vaccine technology, but how the politicization of vaccines resulted in a rise of vaccination refusal [1, 2]. This refusal along with the pandemic brought to the forefront the necessity to identify and develop novel antivirals [3]. Along with SARS-CoV-2 and other listed priority viruses with pandemic potential, the WHO provided a list of prototype pathogens selected for their potential to cause public health emergencies [4]. These lists include viruses from 27 viral families further emphasizing the need to invest in antiviral development.

Among the list of priority and prototype viruses, members from the *Togoviridae*, *Phenuiviridae*, *Paramyxoviridae*, and *Flaviviridae* are neurotropic [5]. Neurotropic viruses are characterized by their ability to infect the central nervous system (CNS), which in many cases results in neuroinflammation that clinically presents as myelitis, meningitis, and encephalitis [6]. Patients may also suffer from post-viral neurological sequelae that includes clinical manifestations including permanent cognitive dysfunction that increase morbidity [7]. Growing evidence further links CNS infections and neurodegenerative diseases, likely promoted via mechanisms induced during viral infection, like neuroinflammation and oxidative stress [8–10]. For many of these neurotropic viruses there are no clinically approved antivirals that target them once they entered the CNS [5, 11].

Tick-borne encephalitis virus (TBEV) is an *Orthoflavivirus* responsible for one of the common tick-borne diseases in Europe, tick-borne encephalitis [12, 13]. Despite the availability of several inactivated vaccines, vaccination rates in endemic areas are poor, incidence is rising, and with no approved antiviral, the population is vulnerable to developing severe disease [12]. The disease caused by TBEV infection is biphasic and neurological symptoms are associated with the second phase, which includes encephalitis and meningitis [14]. TBEV infection of the CNS is further confirmed by immunohistochemical detection of viral antigen in brain samples from fatal human cases [15, 16]. However, the entry route(s) utilized by TBEV to access the CNS are not fully elucidated. One proposed access point to the CNS is the blood-brain barrier (BBB) [17]. The BBB is comprised of brain endothelial cells that express tight junctions and efflux transporters that together with the lack of fenestrations leads to a tightly regulated passage of molecules [18, 19]. Completing the main BBB unit are the pericytes and the astrocytes that support endothelial cell function and regulate immune responses [20, 21].

The BBB may serve as a critical access point for neurotropic viruses, but it is also a hindrance in the CNS dissemination of compounds [22, 23]. With the obstacle the BBB poses, intranasal, intraparenchymal, and drug administration via the blood-cerebral spinal fluid (CSF) barrier are other proposed drug delivery avenues into the CNS [24]. However, the BBB is the largest interface of blood-brain exchange [18]. The extent of the BBB would allow for antivirals to penetrate various parts of the brain. This is an essential feature for treatment, since neurotropic viruses may have widespread distribution in the brain during infection [16, 25]. Multiple antiviral drugs have been developed for antiretroviral therapy against the human immunodeficiency virus (HIV), which can enter the brain and contribute to neurocognitive disorders and neurological comorbidities. BBB-mediated uptake of anti-HIV drugs into the brain is well-described and is influenced by multiple factors including their physicochemical properties, and diverse uptake and efflux mechanisms [26]. In addition, their susceptibility to BBB metabolism may affect further their capacity to enter the brain [27]. These extensively studied antiviral drugs underscores the need to develop well-characterized and validated experimental systems for the evaluation of functional antiviral penetrance through the BBB.

To help advance the development of antiviral drugs against neurotropic viruses, we established an experimental cell culture model of the human BBB. This model consists of primary human brain microvascular endothelial cells (HBMEC) as well as pericytes (HBMVP) co-cultured on transwell inserts. The BBB model was integrated into an experimental antiviral drug screening platform referred to as BASA (BBB-permeable antiviral screening assay) and validated with the use of clinical anti-HIV drugs. Inclusion of human astrocytes to the BBB model revealed that TBEV targets astrocytes without BBB disruption. Through the application of BASA, we identified a novel BBB-permissive TBEV antiviral. The versatility of our assay/model may not only assist in the discovery of CNS-penetrating antivirals, but it may provide further insight to the role of the BBB during viral infections of the brain.

## Materials and methods

### Cell culture

Primary HBMEC, originally purchased from Cell Systems (ACBRI 376), were a kind gift from Professor Ana Rodriguez. Primary HBMVP (PELOBiotech) were provided by Dr. Bugert (Bundeswehr Institute of Microbiology). HBMEC and HBMVP were grown in advanced Dulbecco’s modified eagle medium: nutrient mixture F12 (DMEM/F12, Gibco) with 5% fetal bovine serum (FBS, Gibco), 1X GlutaMAX (Gibco), 1% penicillin/streptomycin (P/S, Gibco), and 1% endothelial growth factor supplement (ECGS, Sciencell) or 1% pericyte growth factor supplement (PGS, Sciencell), respectively. For all experiments HBMEC and HBMVP were used to a maximum number of 15 passages. Glioblastoma cell line, U251 MG, was grown in DMEM/F12 with 1X GlutaMAX, 10% FBS, and 1% P/S. LC5-RIC, HIV reporter cell line, HEK293T, Vero E6, and A549 cells were grown in Dulbecco’s modified eagle medium (DMEM) high glucose (Gibco) with 10% FBS, 1% GlutaMAX, and 1% P/S. LC5-RIC were under selection pressure with the addition of 0.74 mg/ml geneticin (Gibco) and 0.125 mg/ml hygromycin (Gibco) to the medium, as previously described [28, 29]. All cells were grown at 37 °C and 5% CO_2_.

### HNSC.100 culture and differentiation

HNSC.100 were grown in DMEM/F12 with 1X GlutaMAX, 0.5% FBS, 1% P/S, 5mM HEPES buffer solution (Gibco 15630-056), 1% bovine serum albumin (P, Sigma-Aldrich A7906), 1X N2 supplement (Gibco 17502-048), 20 ng/mL fibroblast growth factor (FGF, PeproTech 10018B), and 20 ng/mL epidermal growth factor (EGF, PeproTech AF10015) (proliferation media). Cells were maintained in poly-L-lysine (PLL, Sigma P4707)-coated T75 cell culture flasks. For differentiations, HNSC.100 were seeded in proliferation media at 225,000 cells per well in PLL-coated 24-well plates. Once cells reached 80–90% confluency, approximately 24 h after seeding, cells were washed 1X with phosphate-buffered saline (PBS). The cells were cultured in proliferation media lacking FGF and EGF (differentiation media) with media changes every 2–3 days for 14 days. HNSC.100 progenitor and differentiated cells were maintained at 37 °C and 5% CO_2_.

## Compounds

Stock solutions of antiretrovirals nevirapine (NEV, Sigma SML0097) and saquinavir (SAQ, Biomol Cay9001893) were dissolved in dimethyl sulfoxide (DMSO, Sigma) at 10 mM. Enfuvirtide (T20, Sigma SML0934) 5 mM stock solutions were prepared with DMSO. A 2.5 mM abacavir (ABC, Biomol Cay14746) stock solution was prepared with PBS. Ribavirin (RBV, Biomol LKT-R3205) 100 mM stock solution was prepared with DMSO. Compound 3d 10 mM stock solutions prepared in DMSO and the details on its synthesis were previously described [29]. All compound stock solutions were stored at -20 °C.

### Virus production

HIV-1 (HIV-1_LAI_ laboratory isolate) stocks were generated by the transfection of HEK293T cells with the proviral plasmid pLAI.2 and titrated as previously described [30, 31].

TBEV Hypr was provided by Dr. Bugert (Bundeswehr Institute of Microbiology). TBEV was propagated in Vero E6 cells cultured in DMEM high glucose with 10% FBS, 1% GlutaMAX, and 1% P/S. Viral titers were determined in A549 cells by the TCID50 (50% tissue culture infectious dose) assay and utilizing the Reed-Muench method for calculations [32]. All work involving HIV-1 and TBEV was performed in the biosafety level 3 (BSL3) facility at the Helmholtz Center Munich.

### Establishment of in vitro human BBB transwell model

Transwell inserts with 0.4 μm pores (Corning 3470) were coated with 40 µg/ml of rat collagen type I (Sigma C3867) on the apical side. HBMVP were seeded on the basal side at 5,000 cells/insert in cell culture media and incubated in an inverted position for 24 h. Inserts were returned to the 24-well plate containing HBMVP cell culture media. HBMEC were then seeded on the apical side of the insert at 15,000 cells/insert in HBMEC cell culture media and incubated for 48 h. At this HBMEC seeding density confluent monolayers are likely to be achieved since stable cellular impedance of HBMEC monocultures is observed by 48 h (Supplementary Fig. 1). All incubation steps were at 37 °C and 5% CO_2_.

### TEER measurements

Following the coating and seeding of HBMVP as described above, transwell inserts were transferred to the cellZscopeE device (nanoAnalytics) containing HBMVP cell culture media in the bottom electrodes. HBMEC (15,000/insert) or HBMEC cell culture media were added to the apical side of inserts, and the top electrodes were assembled back with the bottom electrodes. Transendothelial electrical resistance (TEER) measurements were monitored for 48 h at 37 °C and 5% CO_2_.

### Dextran permeability assay

Following the experimental endpoint, cell culture media was removed, and transwell inserts were transferred to a new 24-well plate containing 600 µl of DMEM/F12 with no phenol red (Gibco). Inserts were incubated with 1 mg/mL (100 µl) of FITC-dextran (70,000 MW, Invitrogen) for 1 h at 37 °C and 5% CO_2_. Transwell inserts were then removed, and fluorescence measurements of the basal supernatants were acquired with the Tecan SPARK or infinite M200 plate reader (Tecan). The following acquisition settings were used: excitation wavelength, 485 nm; excitation bandwidth, 9 nm; emission wavelength, 535 nm; emission bandwidth, 20 nm; integration time, 20 µs.

### Immunofluorescence

For the detection of CD31 and neural/glial antigen 2 (NG2), HBMEC (15,000/well) and HBMVP (10,000/well) were seeded in 96-well plates (Greiner Bio-One) and incubated for 24 h at 37 °C and 5% CO_2_. Cell culture media was removed and cells washed once with PBS. Cells were fixed for 30 min with 4% paraformaldehyde (PFA, Santa Cruz Biotechnology) at room temperature. Following two washes with PBS, cells were permeabilized with 0.25% Triton X-100 (Bio-Rad) in PBS for 10 min at room temperature. Cells were washed three times with PBS and blocked for 45 min at room temperature with 5% goat serum (Gibco) in PBS. The blocking solution was removed, and cells were incubated with primary antibodies overnight at 4 °C. All antibodies were diluted in PBS with 0.1% goat serum and all the antibodies used along with the dilutions are listed in Supplementary Table 2. Following the incubation with primary antibodies, cells were washed twice with PBS and incubated with secondary antibodies for 2 h at room temperature. Following two washes with PBS, cells were incubated with a 1:10,000 dilution of Hoechst 33,342 (Invitrogen) for 5 min at room temperature. Fluorescence images were acquired on the LSM 980 (Zeiss) using a 10x objective. Further image processing was conducted with the ZEN microscopy software (Zeiss). For the CD31 primary antibody used in this study, diffuse cytoplasmic staining may be observed, which is also reported by the manufacturer.

For the detection of *Flavivirus* envelope (E) protein in HBMEC and HNSC.100-differentiated astrocytes, fixation and antibody staining proceeded as described above. With the exception that volumes were adjusted for a 24-well plate format.

### Flow cytometry

For CD31 and NG2 detection, confluent cultures of HBMEC and HBMVP were harvested with TrypLE Express (Gibco) and washed with 20% FBS in PBS. After centrifugation, cells were incubated with primary antibody staining solutions prepared in PBS with 0.1% BSA for 30 min at room temperature. All antibodies used for flow cytometry are listed in Supplementary Table 2. Following a wash with 0.1% BSA in PBS, cells were incubated with secondary antibody or other conjugated antibody solutions prepared in PBS with 0.1% BSA for 30 min at room temperature. After a wash, cells were fixed with 4% PFA for 15 min at room temperature and then washed once with 0.1% BSA in PBS. Cells were resuspended in PBS with 0.1% BSA and processed using a CytoFLEX S flow cytometer (Beckman Coulter). Processed samples were analyzed by FlowJo v10 (BD Biosciences).

After each TBEV infection timepoint, HBMEC, HBMVP, and U251 MG were harvested with TrypLE Express and washed with 20% FBS in PBS. Cells were fixed with 4%PFA for 15 min at room temperature and then washed with 3% BSA in PBS. Cells were permeabilized with 0.25% Triton X-100 in PBS for 10 min at room temperature. Following a wash with 3% BSA in PBS, cells were incubated with antibody solutions prepared in PBS with 0.1% BSA for 30 min at room temperature. Cells were washed once and then resuspended in 0.1% BSA. Samples were processed using CytoFLEX LX flow cytometer (Beckman Coulter) and analyzed by FlowJo v10 (BD Biosciences).

HNSC.100-differentiated astrocytes after each TBEV infection timepoint were harvested as described above. Astrocytes were then stained for 20 min at room temperature with ViaKrome 808 fixable viability dye (Beckman Coulter) prepared as per the manufacturer’s instructions. Cells were washed once with 3% BSA in PBS. Fixation, permeabilization, antibody staining, and sample processing proceeded as described above.

### Quantitative reverse transcription polymerase chain reaction (RT-qPCR)

Cells were harvested at the indicated timepoints, and RNA was extracted using a NucleoSpin RNA kit (Macherey-Nagel) as per the manufacturer’s instructions. RNA was eluted in a volume of 50 µL. For cDNA synthesis, PrimeScript^™^ RT Master Mix (TaKaRa) was used according to the manufacturer’s instructions. For quantitative PCR 25 ng of cDNA was used and performed with the QuantStudio 5 system (ThermoFisher Scientific), using PowerUp^™^ SYBR^™^ Green Master Mix (Applied Biosystems). To detect TBEV viral RNA we used previously published primer sequences [33]. All primer sequences used are listed in Supplementary Table 3. Data were analyzed with the second derivative maximum method. The relative amount was calculated by the 2^(−ΔΔCt)^ method using *ribosomal protein lateral stalk subunit P0* (*RLPL0)* as a reference gene.

### Monoculture TBEV infections

HBMEC and HBMVP were seeded in 24-well plates at 55,000 cells/well and 50,000 cells/well, respectively. After 24 h, the media was removed. TBEV Hypr inoculum prepared in the HBMEC or HBMVP cell culture media was added to cells at a multiplicity of infection (MOI) of 0.1, 1, and 10. Virus inoculum was not removed in order to prevent mechanical damage of monolayer integrity. At 1-, 2-, and 3-days post infection (dpi) cells were harvested for flow cytometry, RT-qPCR, or plates fixed for immunofluorescence staining. On day 14 of differentiation, media was removed and TBEV Hypr inoculum prepared in DMEM/F12 with 1X GlutaMAX (Gibco) to an MOI of 0.1, 1, and 10 was added to HNSC.100-differentiated astrocytes. After a 1-hour incubation at 37 °C and 5% CO_2_, viral inoculum was removed and fresh differentiation media was added to cells. At 1-, 2-, and 3-dpi cells were harvested for flow cytometry, RT-qPCR, or plates fixed for immunofluorescence staining.

### In vitro BBB transwell model TBEV infections

The BBB transwell model with HBMEC and HBMVP was prepared as described above and referred to as BBB. Empty collagen-coated transwell inserts were referred to as No BBB. The media of day 14 HNSC.100-differentiated astrocytes was replaced with differentiation media supplemented with 0.5% PGS. Inserts were combined with astrocyte cultures and fresh HBMEC cell culture media was added to the apical side of inserts. After a 30-minute incubation at 37 °C and 5% CO_2_, TBEV Hypr inoculum at a MOI of 0.1 prepared in HBMEC cell culture media was added to the apical side of inserts. The cultures were incubated at 37 °C and 5% CO_2_ for 1, 2, and 3 days. For each timepoint, BBB inserts were collected for the dextran permeability assay and HNSC.100-differentiated astrocytes harvested for flow cytometry and RT-qPCR.

### BBB-permeable antiviral screening assay (BASA), method-linked Fig. 1

**Fig. 1:**
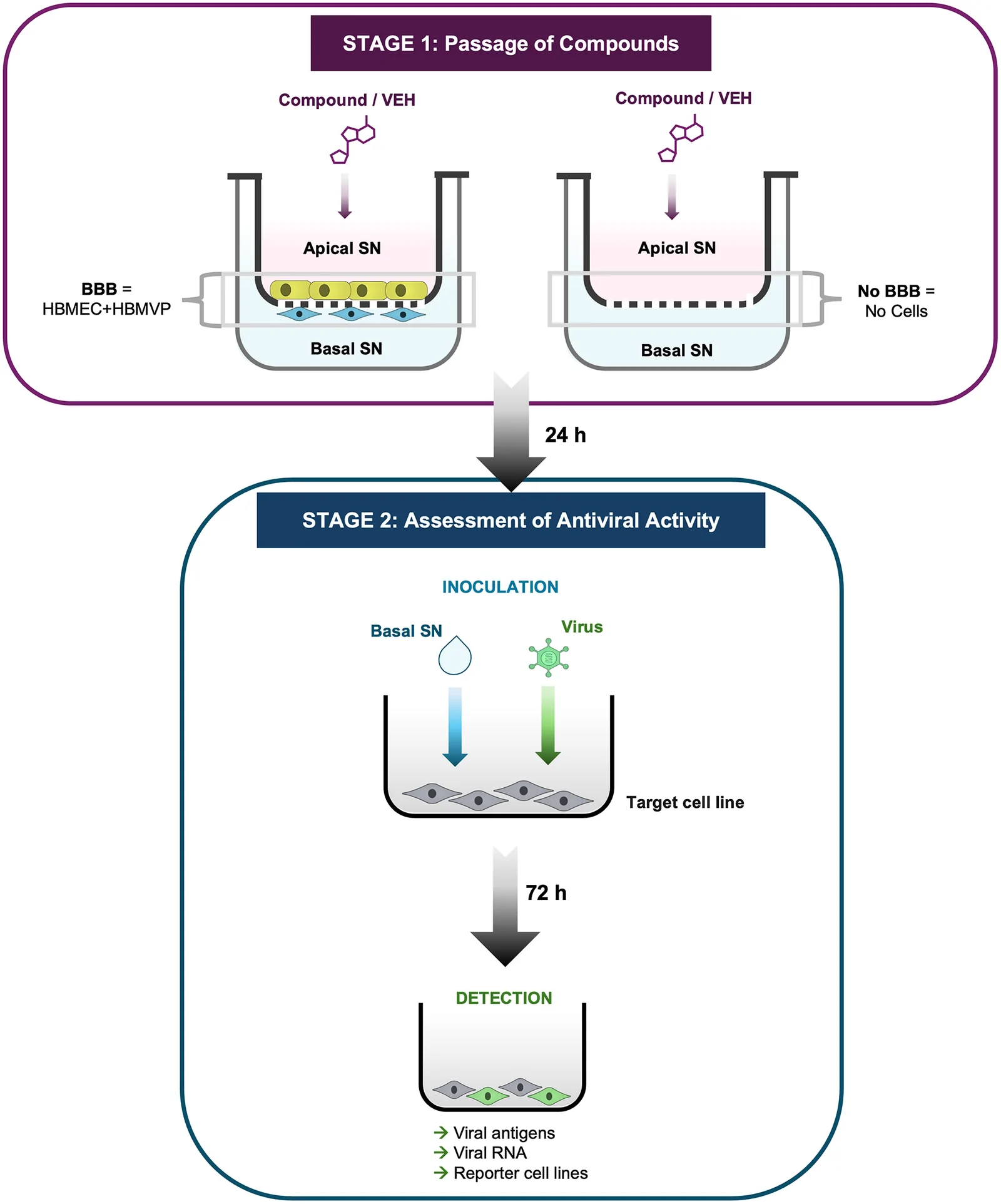
BASA: A 2-stage assay to functionally screen for BBB-permeable antivirals. Stage 1: Compound of interest and the respective vehicle (VEH) are added to HBMEC+HBMVP (BBB)-containing or empty transwell inserts (No BBB). The concentrations of compounds in the apical supernatant (SN) are adjusted based on the desired inhibitory concentration for the basal SN. Inserts are then incubated for 24-hours (h). Stage 2: The target cell line is incubated with virus inoculum and basal SN for 72 h. Detection of viral infection in target cells can be determined by the presence of viral antigens (cytometric analysis), viral RNA (RT qPCR), and if available reporter cell lines (fluorometric measurement). The detailed set-up of BASA is described in material and methods

Graphical description of BASA is illustrated in Fig. 1. **STAGE 1**: After setting up BBB transwell models (BBB) as described above and empty collagen-coated transwell inserts (No BBB), the basal media was replaced with HBMVP cell culture media, but with 0.5% instead of 1% PGS. The media from the apical side of inserts was replaced with compound or the respective vehicle dilutions. All compound dilutions were prepared in HBMEC cell culture media. Considering the volume differences and diffusion flux, compound concentrations added on the apical side were 3.4x higher than the desired basal supernatant concentrations. To achieve IC_90_ in the basal supernatant, the following concentrations were used for the antiretrovirals: NEV (107.44 nM), ABC (26.86 µM), SAQ (126.14 nM), and T20 (25.5 nM). To achieve the double IC_50_ in the basal supernatant, RBV was added at a concentration of 804.4 µM and compound 3d at 306 µM. After a 24-hour incubation at 37 °C and 5% CO_2_, inserts were removed and basal supernatants collected. BBB inserts exposed to RBV and D12 were processed for the dextran permeability assay. **STAGE 2**: 24 h prior to supernatant collection target cells were seeded for antiviral testing. For HIV, LC5-RIC were seeded in 96-well plates at 20,000 cells/well. For TBEV, U251 MG were seeded in 24-well plates at 100,000 cells/well. Cell culture media was removed and basal supernatants collected from step 1 were added to the target cells, followed by the addition of viral inoculum at a MOI of 0.5 (HIV-1) or MOI 0.1 (TBEV Hypr). Cells were then incubated for 72 h at 37 °C and 5% CO_2_. Antiviral effects in HIV-infected LC5-RIC were fluorometrically measured as previously described using a Tecan SPARK plate reader (Tecan) [28]. For the detection of TBEV infection, U251 MG were harvested then processed for RT-qPCR and flow cytometry as described above. Relative infection levels were determined based on the viral infections measured in target cells exposed to vehicle-treated basal supernatants.

### Graphics

All graphics were generated from images purchased from Simplified Science Publishing and the following downloaded NIH BIOART images: NIAID Visual & Medical Arts. (10/7/2024). Brain Lateral. NIAID NIH BIOART Source.bioart.niaid.nih.gov/bioart/60; and NIAID Visual & Medical Arts. (3/20/2025). Zika Virus. NIAID NIH BIOART Source. bioart.niaid.nih.gov/bioart/604.

### Statistical analysis

Statistical analyses were performed on experimental data from at least three biological replicates (n) in Prism 10 (Graphpad Software). The Shapiro-Wilk test was used to test whether the data followed normal distribution. If the data were not normally distributed, the following nonparametric tests were used: the Wilcoxon test (paired data), the Mann-Whitney test (unpaired data), or for more than two groups the Kruskal-Wallis test. For normally distributed data, an unpaired t test or paired t test (two groups), one-way analysis of variance (ANOVA) (> two groups, 1 variable) or two-way ANOVA (> two groups, two variables) were used. The exact statistical tests used are listed in the figure legends, and the significance levels denoted with asterisks: *, *P* < 0.05; **, *P* < 0.01; ***, *P* < 0.001; ****, *P* < 0.0001.

## Results

### Set-up of a functional BBB-permeable antiviral screening assay (BASA)

In vitro models to predict the BBB permeability of drugs are established but they are largely focused on measuring the amount of drug that crosses the BBB [34–36]. For the discovery of antivirals against neurotropic viruses, it is not only critical they cross the BBB but also remain active, since the BBB is a metabolically active barrier that may affect drug activity [27].

To identify active BBB-permeable antivirals, we established a two-stage assay combining our transwell BBB model with antiviral testing. In stage 1, the compound of interest is added to the apical side of the transwell insert containing HBMEC+HBMVP (BBB) or no cells (No BBB) (Fig. 1, Stage 1). The empty transwell insert (No BBB) serves as a control for the maximum permeation of the compound. In addition, BBB and No BBB transwell inserts incubated with vehicle (VEH) are included as negative controls. After 24 h the basal supernatant is collected. Subsequently, basal supernatant samples and virus inoculum are simultaneously added to a viral target cell line (Fig. 1, Stage 2). The selection of the target cell line and the incubation period (72 h) can be adjusted to the virus of interest. After viral inoculation, infection levels in the target cells can be determined by the presence of viral antigens, viral RNA, or the fluorometric changes of a suitable reporter cell line (Fig. 1, Stage 2). The basal supernatants collected from the No BBB transwells serve as references to the maximum expected antiviral activity. The detailed set-up of the assay is described in material and methods. Through evaluating the antiviral activity in the basal supernatants, we can identify BBB-permeable compounds that remain active to exert their antiviral effects. Overall, this two-stage assay provides a functional assessment of candidate antivirals that can be tailored to the virus of interest.

### In vitro human BBB established in transwell inserts

**Fig. 2:**
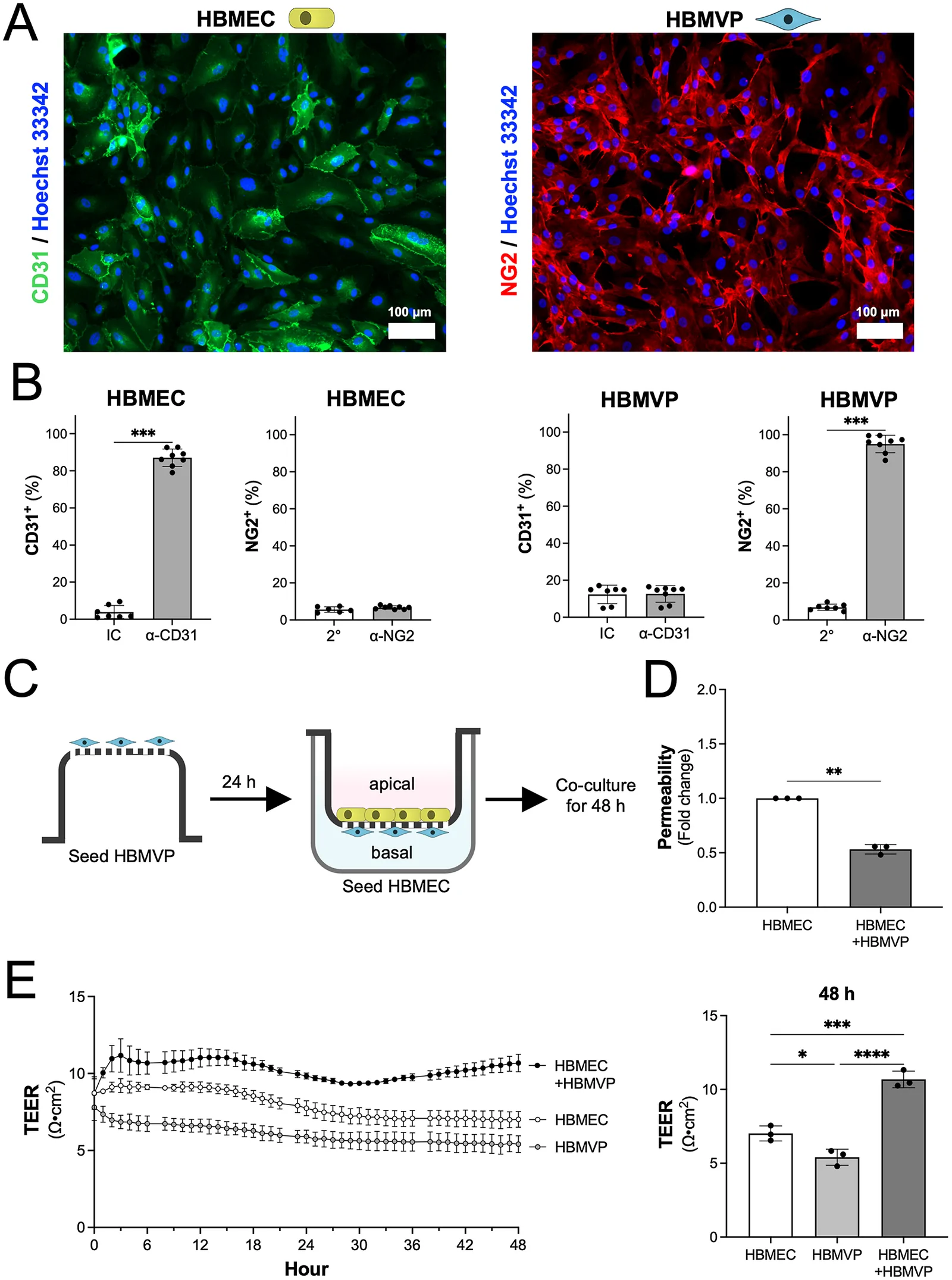
In vitro BBB transwell model established with primary HBMEC and HBMVP. (**A**) Immunofluorescence images of CD31 positive (green) HBMEC and neural/glial antigen 2 positive (NG2, red) HBMVP. Nuclei were stained with Hoechst 33,342 (blue). (**B**) Cytometric measurement of CD31 and NG2 surface expression. Isotype control (IC) and secondary (2º) antibodies were included as controls. (**C**) Schematic of BBB establishment in transwell inserts. HBMVP were incubated on the basal side of an inverted insert for 24 h (h) followed by the addition of HBMEC on the apical side. Cells were co-cultured for 48 h. (**D**) After 48 h, barrier integrity of monocultures of HBMEC or HBMEC+HBMVP co-culture was determined by measuring the leakage of 70 kDa FITC-dextran. Data are expressed as fold change relative to the fluorescence values of HBMEC monocultures (HBMEC). (**E**) Transendothelial electrical resistance (TEER) measurements in transwell inserts following the addition of HBMEC (hour = 0). TEER in HBMEC only, HBMVP only, and HBMEC+HBMVP transwell inserts was monitored for 48 h. Bar graph of the 48 h timepoint is shown. Data presented as the mean ± SD (*n* = 4 (**B**) and *n* = 3 (**D**, **E**)). Statistical significance was determined by Mann-Whitney test (**B**), Paired t-test (**D**), or Ordinary one-way ANOVA with Tukey’s multiple comparison test (**E**). *p ≤ 0.05, **p ≤ 0.01, ***p ≤ 0.001, ****p ≤ 0.0001. Non-significant differences were not shown

Key cellular players of the BBB include brain endothelial cells that separate the brain parenchyma from the circulation and pericytes that promote barrier integrity [37]. For our model we used primary human brain microvascular endothelial cells (HBMEC) and primary human brain microvascular pericytes (HBMVP). Microscopy and cytometric analysis revealed unique expression of CD31 and neural/glial antigen 2 (NG2) in HBMEC and HBMVP, respectively (Fig. 2A and B). The unique expression of these previously reported markers offers a clear distinction between HBMEC and HBMVP in our model [38, 39].

In vitro BBB models ranging from organoid to microfluidic models have been established [40–42]. Every BBB model is faced with technical and physiological limitations and selecting the appropriate model is dependent on the scientific question [43–45]. For application as a screening tool, we opted for the transwell insert model that offers a streamline set-up, with the HBMVP located on the basal side and the HBMEC on the apical side of the transwell insert (Fig. 2C), and requiring a set-up time of 72 h. To compare permeability changes in HBMEC/HBMVP co-cultures relative to monocultures we utilized a dextran-based permeability assay. The passage of 70 kilodalton (kDa) FITC-conjugated dextran was decreased in HBMEC and HBMVP co-cultures relative to HBMEC monocultures (Fig. 2D). This significant decrease was also observed with lower molecular weight dextrans (Supplementary Fig. 2). Similarly, transendothelial electrical resistance (TEER) was significantly higher in HBMEC and HBMVP co-culture than in monocultures (Fig. 2E). These data demonstrate that the co-culture of HBMEC and HBMVP is important in promoting a tighter barrier.

### Antiretrovirals validate BASA, a BBB-permeable antiviral screening assay

Human immunodeficiency virus (HIV) is one of a few neurotropic viruses with clinically approved antivirals. Anti-HIV drugs showcase antiviral drug development, with extensive academic and industry efforts culminating in the development of over 25 U.S. Food and Drug Administration (FDA) approved clinical drugs [46]. Due to these efforts, anti-HIV drugs are well-characterized and the CNS penetration efficiency for many of them is known [26]. The publication of CNS penetration effectiveness (CPE) scores for anti-HIV drugs provides a ranking system based on pharmacological and clinical data [47]. A high CPE score indicates the drug has greater penetration into the CNS than one with a lower CPE score.

**Fig. 3:**
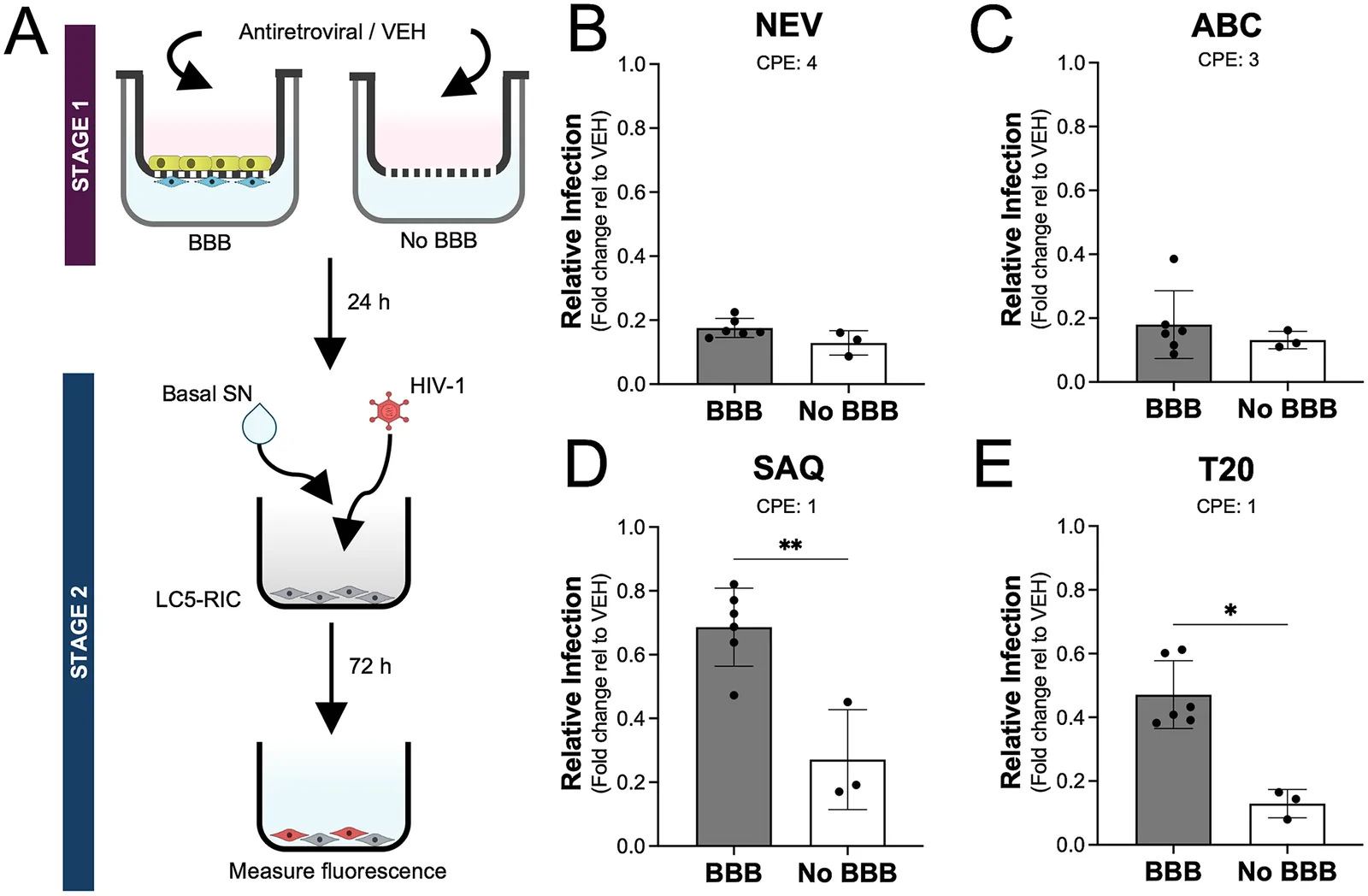
Differences in antiviral activity distinguish highly BBB-permeable nevirapine and abacavir from less permeable anti-HIV drugs. (**A**) BASA adjusted for HIV-1 infection. Stage 1: anti-HIV drugs or vehicle (VEH) were added to the apical side of inserts containing HBMEC+HBMVP (BBB) or no cells (No BBB). Stage 2: After 24 h (h), basal supernatants (SN) were simultaneously added with HIV-1 inoculum (MOI 0.5) to LC5-RIC, a HIV reporter cell line. HIV infection in LC5-RIC was fluorometrically measured after 72 h. (**B**-**E**) Effects of basal SN from nevirapine (NEV, B), abacavir (ABC, **C**), saquinavir (SAQ, **D**), or enfuvirtide (T20, **E**)-exposed inserts on HIV-1 infection in LC5-RIC. 2010 CNS penetration effectiveness (CPE) scores are listed [47]. Infection levels are expressed relative to fluorescence values measured in LC5-RIC incubated with HIV-1 and basal SN from VEH-treated inserts. Data presented as the mean ± SD (*n* = 3). Statistical significance was determined by Unpaired t-test (D) or Mann-Whitney test (E). *p ≤ 0.05, **p ≤ 0.01. Non-significant differences were not shown

To validate BASA as a tool for identifying active BBB-permeable antivirals, we compared the antiviral activity of anti-HIV drugs with low and high CPE scores. Nevirapine (NEV) and abacavir (ABC) along with saquinavir (SAQ) and enfuvirtide (T20) were selected as representative drugs with high and low CPE scores, respectively (Fig. 3) [47]. These drugs were added to the apical side of the BBB and No BBB transwell inserts, as previously described for stage 1 of BASA (Fig. 1). For stage 2, LC5-RIC, an HIV reporter cell line [28], was selected as the target (Fig. 3A). Following a 72-hour incubation with the basal SN and HIV-1 inoculum, infection levels were fluorometrically measured (Fig. 3A, Stage 2). The expected maximal antiviral activity in the No BBB basal SN corresponds to the drugs’ IC_90_ calculated from IC_50_ curves, which are within range of published reports (Supplementary Fig. 3A-D) [28, 48, 49].

We observed the basal SN from NEV- or ABC-exposed BBB inserts reduced HIV infection to similar levels observed when No BBB is present (Fig. 3B and C). This suggests NEV and ABC can permeate across our in vitro BBB and reduce HIV infection, which correlates with their high CPE scores. Furthermore, the ability of these compounds to cross is not a consequence of cytotoxic effects since the concentrations used do not reduce the viability of HBMEC and HBMVP (Supplementary Fig. 4A and 4B). However, SAQ and T20 do not readily cross our in vitro BBB since HIV infection was significantly higher in LC5-RIC that received BBB basal SN in comparison to No BBB (Fig. 3D and E). These results agree with the low CPE scores for SAQ and T20. The retention of SAQ and T20 is further evidenced by the significantly lower drug concentrations calculated in the basal SN of BBB inserts compared to No BBB inserts (Supplementary Fig. 5C and 5D). As predicted from the drug concentrations added to the apical side of inserts, the calculated concentrations for all No BBB basal SN are within the range of the drugs’ IC_90_ (Supplementary Fig. 5). Along with further validating the BASA set-up, the parallel application of drug titration curves may offer a means to quantitatively estimate the retention of a compound via the calculation of the active drug concentration in the basal SN. Overall, the data presented here behave in accordance with the published CPE scores and demonstrate that BASA can functionally distinguish between antivirals with high and low potential of crossing the BBB.

### Primary HBMEC and HNSC.100-differentiated astrocytes are susceptible to TBEV infection

Tick-borne encephalitis virus (TBEV) is a neurotropic *Orthoflavivirus*, capable of causing neurological symptoms that include encephalitis [13]. Unlike HIV, there are no clinically approved antivirals for TBEV and no compounds that can target the virus once it breaches into the CNS. The treatment of patients relies completely on supportive care [50].

**Fig. 4:**
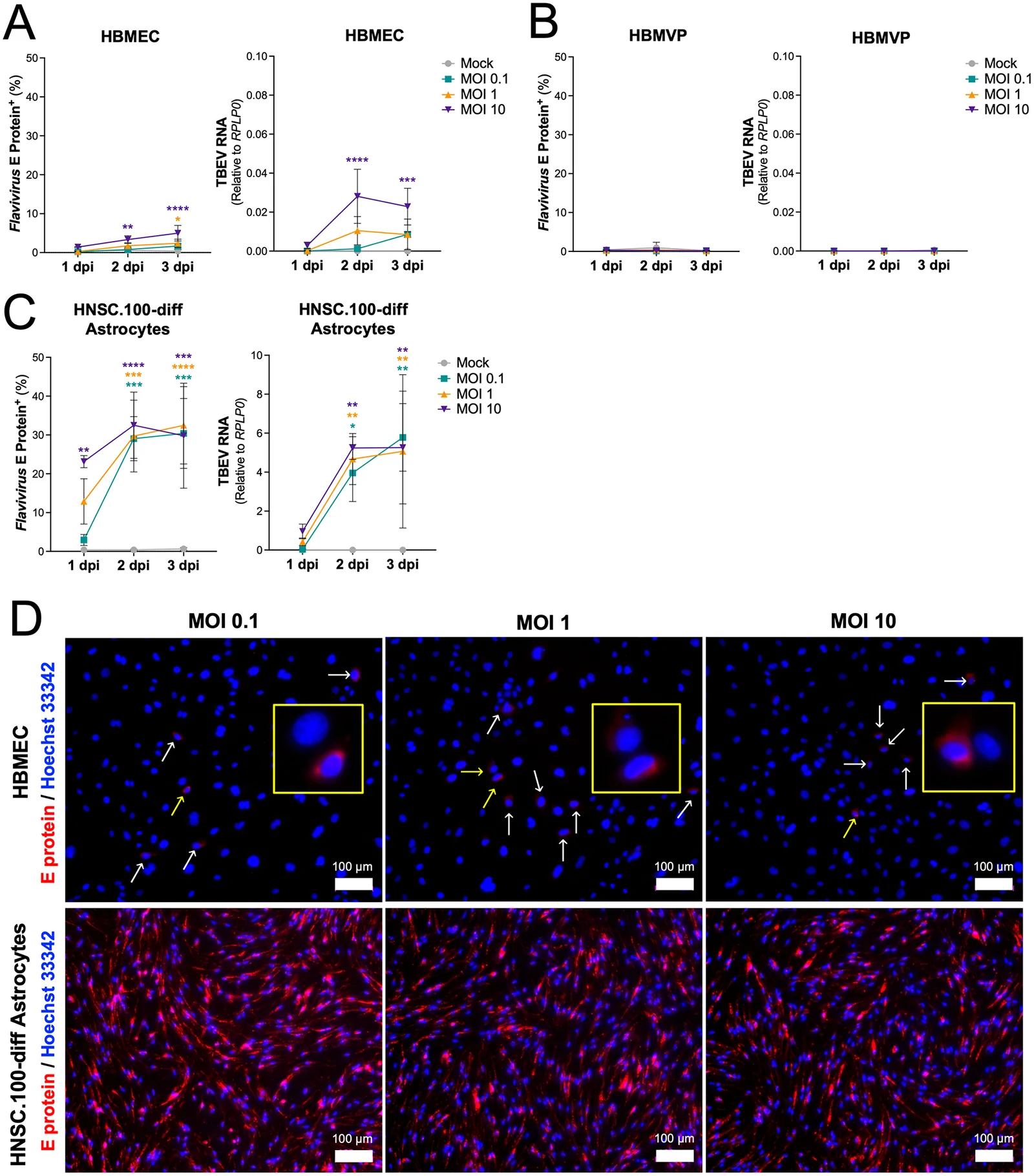
TBEV infects primary HBMEC and HNSC.100-derived astrocytes. (**A**-**C**) Monocultures of HBMEC (**A**), HBMVP (**B**), and HNSC.100-differentiated (diff) astrocytes (**C**) were inoculated with different MOI of TBEV Hypr. Cells were harvested at 1-, 2-, and 3-days post-infection (dpi). For each timepoint, infection was detected by flow cytometry with an antibody targeting the *Flavivirus* envelope (E) protein. The percentage of E positive cells is shown (left panels). Further confirmation of infection was determined by TBEV RNA levels shown relative to the housekeeping gene, *RPLP0* (right panels). (**D**) Immunofluorescence images of TBEV-infected HBMEC and HNSC.100-differentated astrocytes at 3-dpi. Cells were stained with ⍺-*Flavivirus* E protein (red) and Hoechst 33,342 (nuclei, blue). For HBMEC panels arrows indicate cells positive for E protein staining and an enlarged frame of representative positive cells is shown. Data presented as the mean ± SD (*n* = 3). Statistical significance was determined by 2way ANOVA with Tukey’s multiple comparisons test. *p ≤ 0.05, **p ≤ 0.01, ***p ≤ 0.001, ****p ≤ 0.0001. Non-significant differences were not shown

Before screening potential antivirals, we determined whether the cells in our BBB model are targets for TBEV infection. When HBMEC monocultures were inoculated with TBEV Hypr, a highly pathogenic strain of TBEV [51], less than 5% of HBMEC stained positive for the *Flavivirus* envelope (E) protein even at the highest multiplicity of infection (MOI) tested (Fig. 4A). Similarly, TBEV RNA levels were low with only significant levels observed in MOI 10 at 2- and 3-days post-infection (dpi) (Fig. 4A). The low percentage of E positive HBMEC was confirmed by immunofluorescence (Fig. 4D). At higher magnification, perinuclear staining could be observed, which was previously shown [52] (Supplementary Fig. 6). This staining could be in line with endoplasmic reticulum (ER) localization where *Flaviviruses* are described to form replication complexes [53]. Low TBEV infection in HBMEC has been previously described [52]. Interestingly, we observed that even at MOI 10 TBEV does not disrupt HBMEC monolayer integrity (Supplementary Fig. 7A). This contrasts with the effects of exogenously added TNF-α that transiently disrupts HBMEC monolayer integrity 6 h after addition (Supplementary Fig. 7B). Aside from supporting BBB integrity, pericytes are described to be important cellular players during viral infections [54]. We did not detect *Flavivirus* E protein or TBEV RNA in HBMVP (Fig. 4B).

Astrocytes are integral parts of the BBB that provide barrier support, play a role in inflammatory responses and are potential targets for neurotropic viruses [55, 56]. For the generation of human astrocyte-enriched cell cultures, we differentiated an immortalized human neural stem cell line, HNSC.100 [57], through a 14-day culture in the absence of growth factors. Previous reports demonstrate that under these conditions there is significant upregulation of astrocytic markers, including glial fibrillary acidic protein (*GFAP*), aquaporin 4 (*AQP4*), and S100β (*S100B*) [58–60]. Differentiation-dependent upregulation of the key astrocytic markers GFAP and AQP4 were confirmed for the cultures used in this study (Supplementary Fig. 8).

Flow cytometry analysis revealed that in contrast to HBMEC and HBMVP, a significant percentage of HNSC.100-differentiated astrocytes were E protein positive by 2-dpi after exposure to all tested MOI (Fig. 4C). These observations were further confirmed by immunofluorescent images (Fig. 4D). In addition, TBEV transcript levels were significantly increased by 2-dpi, indicating HNSC.100-differentiated astrocytes are permissive to TBEV infection (Fig. 4C). Despite the high percentage of TBEV-infected cells, TBEV Hypr does not induce a significant increase in the percentage of dead cells (Supplementary Fig. 9A). Since GFAP expression, a marker of activation, is high in the mock-treated HNSC.100-differentiated astrocytes, we did not detect a significant increase during TBEV infection (Supplementary Fig. 9B). Our data is in line with published TBEV infection data of primary and iPSC-derived astrocytes [61–63]. Therefore, HNSC.100-differentiated astrocytes can be used as a target cell line for TBEV.

### TBEV crosses the BBB in the in vitro transwell model without significant effect on barrier integrity

**Fig. 5:**
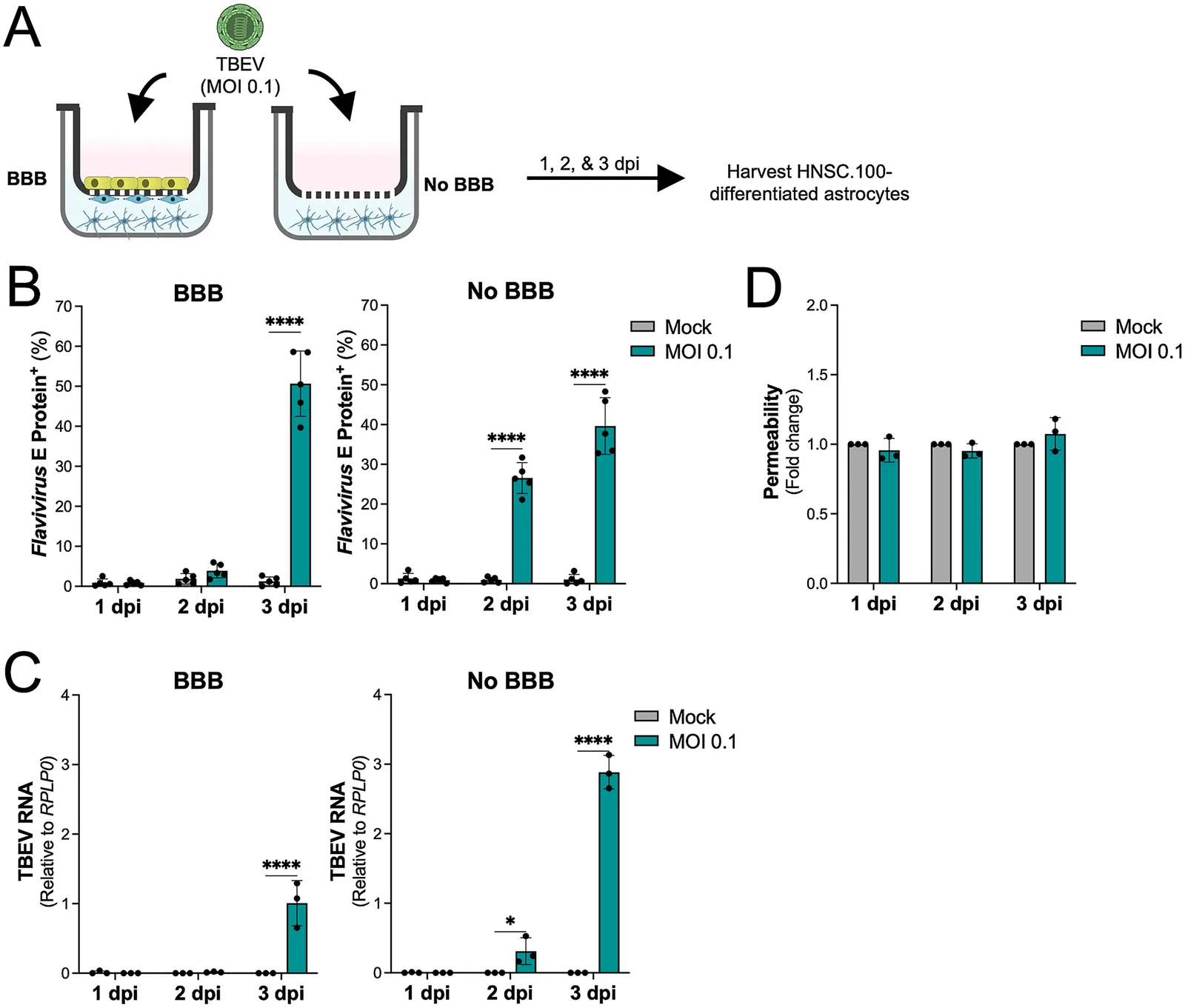
TBEV crosses a barrier of HBMEC and HBMVP to infect HNSC.100-differentiated astrocytes. (**A**) Schematic of the experimental set-up. Inserts containing HBMEC+HBMVP (BBB) or no cells (No BBB) were combined with cultures of HNSC.100-differentiated astrocytes. TBEV Hypr at a MOI of 0.1, relative to HBMEC, was added to the apical side of inserts. HNSC.100-differentiated astrocytes were harvested a 1-, 2-, and 3-days post-infection (dpi). (**B**) Cytometric detection of Flavivirus envelope (**E**) protein positive HNSC.100-differentiated astrocytes. (**C**) TBEV RNA levels in HNSC.100-differentiated astrocytes. Levels shown relative to the housekeeping gene, RPLP0. (**D**) At 1-, 2-, and 3-dpi changes in BBB-insert permeability were determined by measuring the leakage of 70 kDa FITC-dextran. Data expressed as fold change relative to the fluorescence values of mock-treated BBB inserts. Data presented as the mean ± SD (*n* = 3). Statistical significance was determined by 2way ANOVA with Šídák’s multiple comparisons test. *p ≤ 0.05, ****p ≤ 0.0001. Non-significant differences were not shown

A proposed entry point TBEV utilizes to gain access to the CNS is the BBB [64]. However, we observed low to no TBEV infection in HBMEC and HBMVP, respectively (Fig. 4A and B). To determine if TBEV can cross the BBB, we expanded our in vitro BBB model to include astrocytes in a tri-culture set-up. TBEV Hypr was added to the apical side of transwell inserts containing HBMEC and HBMVP (BBB) or no cells (No BBB) with HNSC.100-differentiated astrocytes at the bottom of the transwell plate (Fig. 5A).

As expected from the monoculture infections, a significant percentage of E protein positive HNSC.100-differentiated astrocytes were detected at 2-dpi in the absence of the BBB (No BBB) (Fig. 5B). In the presence of the BBB, a delayed but significant increase in E protein positive HNSC.100-differentiated astrocytes was detected at 3-dpi (Fig. 5B). Similar trends were observed in TBEV transcript levels (Fig. 5C). A significant increase of infectious viral titers was observed at 2- and 3-dpi in the basal supernatants from astrocyte cultures combined with BBB or No BBB inserts (Supplementary Fig. 10). These data indicate HNSC.100-differentiated astrocytes support a productive TBEV infection. However, in the presence of the BBB it is difficult to determine the extent HBMEC contribute to the TBEV titers in the basal supernatant. No significant permeability changes were detected in the BBB inserts, suggesting barrier disruption is not a main mechanism utilized by TBEV to cross our in vitro BBB (Fig. 5D). These data suggest TBEV can cross our in vitro BBB, but the delay in astrocyte infection could be a result of the barrier’s physical restriction or induced host responses.

### Application of BASA reveals novel BBB-permeable antiviral against TBEV

**Fig. 6:**
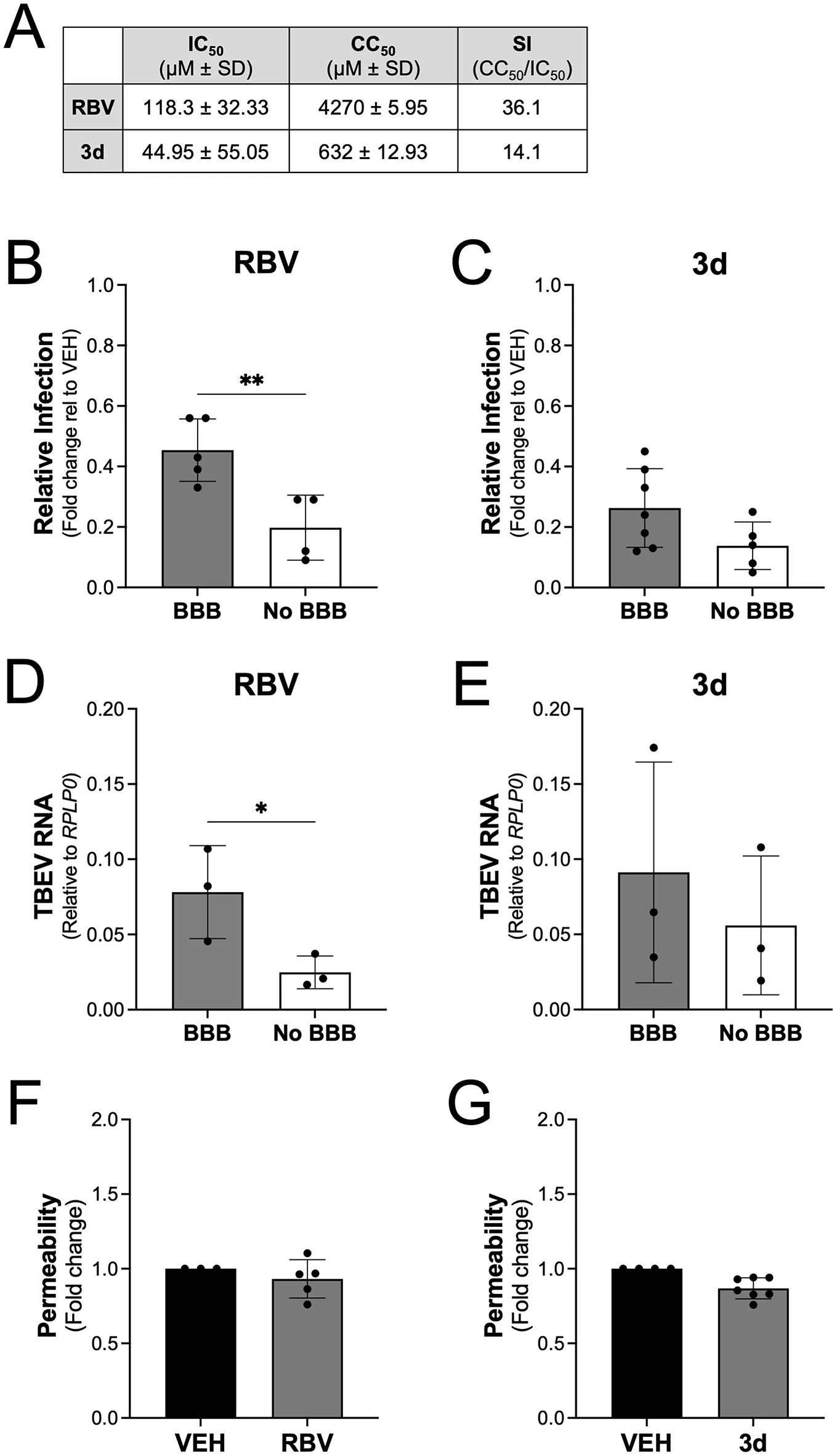
Compound 3d identified via BASA as a BBB-permeable antiviral against TBEV. (**A**) Table with the half-maximal inhibitory concentration (IC50), 50% cytotoxicity concentration (CC50), and selectivity index (SI) of compound 3d (3d) and ribavirin (RBV) determined for TBEV infection in U251 MG cells. (**B**-**G**) Application of BASA. Stage 1: 3d and RBV added to the apical side of HBMEC+HBMVP (BBB) and empty (No BBB) inserts. Stage 2: After 24 h, U251 MG cells were incubated with basal supernatants and TBEV Hypr inoculum (MOI 0.1) for 72 h and TBEV infection determined by the presence of viral antigens and RNA. (**B**, **C**) Cytometric detection of U251 MG cells positive for Flavivirus envelope (**E**) protein at 72-hours post-infection (hpi). Infection levels are expressed relative to E protein positive cells incubated with TBEV and basal supernatants from vehicle (VEH)-exposed BBB or No BBB inserts. (**D**, **E**) TBEV RNA levels in U251 MG cells at 72-hpi. Levels shown relative to the housekeeping gene, RPLP0. (**F**, **G**) Evaluation of permeability changes in BBB inserts after stage 1 measured by the leakage of 70 kDa FITC-dextran. Fold change relative to fluorescence values of VEH-exposed BBB inserts is shown. Data presented as the mean ± SD (*n* = 3 (**B**, **D**-**F**) and n = 4 (**C**, **G**)). Statistical significance was determined by Unpaired t-test (**B**-**E**) or Wilcoxon test (**F**, **G**). *p ≤ 0.05, **p ≤ 0.01. Non-significant differences were not shown

To identify candidate BBB-permeable antivirals against TBEV, we titrated compound 3d, a nucleoside analog shown to inhibit Zika virus (ZIKV) replication [29], in the glioblastoma cell line, U251 MG (Supplementary Fig. 11A). As control, we included ribavirin (RBV) that was previously reported to exert antiviral effects against TBEV [65], and titrated the antiviral in U251 MG (Supplementary Fig. 11B). From these titrations, we determined compound 3d displayed a lower IC_50_ and 50% cytotoxicity concentration (CC_50_) for TBEV infection than RBV (Fig. 6A, Supplementary Fig. 11).

To determine whether compound 3d and RBV crosses the BBB, we applied BASA that was modified to detect TBEV infection (Fig. 1). In brief, stage 1 remained the same with the exception that if the compounds fully permeated across after 24-hours, the expected basal SN concentrations would be double the IC_50_. For stage 2, U251 MG were utilized as the target cell line, and infection was determined by cytometric detection of E protein positive cells at 3-dpi. In U251 MG cells TBEV infection was significantly higher when incubated with basal supernatants from RBV-exposed BBB inserts than basal supernatants from RBV-exposed No BBB inserts (Fig. 6B). A similar trend was observed for viral RNA levels (Fig. 6D). The absence of permeability changes in RBV-exposed BBB inserts compared to vehicle (VEH)-exposed BBB inserts indicate RBV does not disrupt BBB integrity (Fig. 6F). RBV is a highly hydrophilic drug as indicated by its low partition coefficient (logP) (Supplementary Table 1) [66]. A published report further demonstrated that RBV may have poor CNS penetrance [67]. This supports our data that suggests RBV does not efficiently permeate across the BBB.

Unlike RBV, there is no significant difference in TBEV infection between U251 MG cells incubated with basal supernatants from 3d-exposed BBB or No BBB inserts (Fig. 6C). Also, no significant difference is observed in TBEV RNA levels (Fig. 6E). These data suggest compound 3d can cross our in vitro BBB model to exert antiviral effects against TBEV in U251 MG cells. Similarly, the basal supernatants from 3d-exposed BBB or No BBB inserts reduced ZIKV infection in U251 MG to equal levels (Supplementary Fig. 12). This further demonstrates that 3d retains its antiviral activity after passage across the BBB against ZIKV, which was the reported target of 3d [29]. The ability of compound 3d to cross the in vitro BBB is not a consequence of barrier disruption since no significant change in permeability was detected between vehicle (VEH)-exposed or 3d-exposed BBB inserts (Fig. 6G). Here we demonstrate that BASA is a tool that can be applied to discover novel BBB-permeable antivirals.

## Discussion

**Fig. 7:**
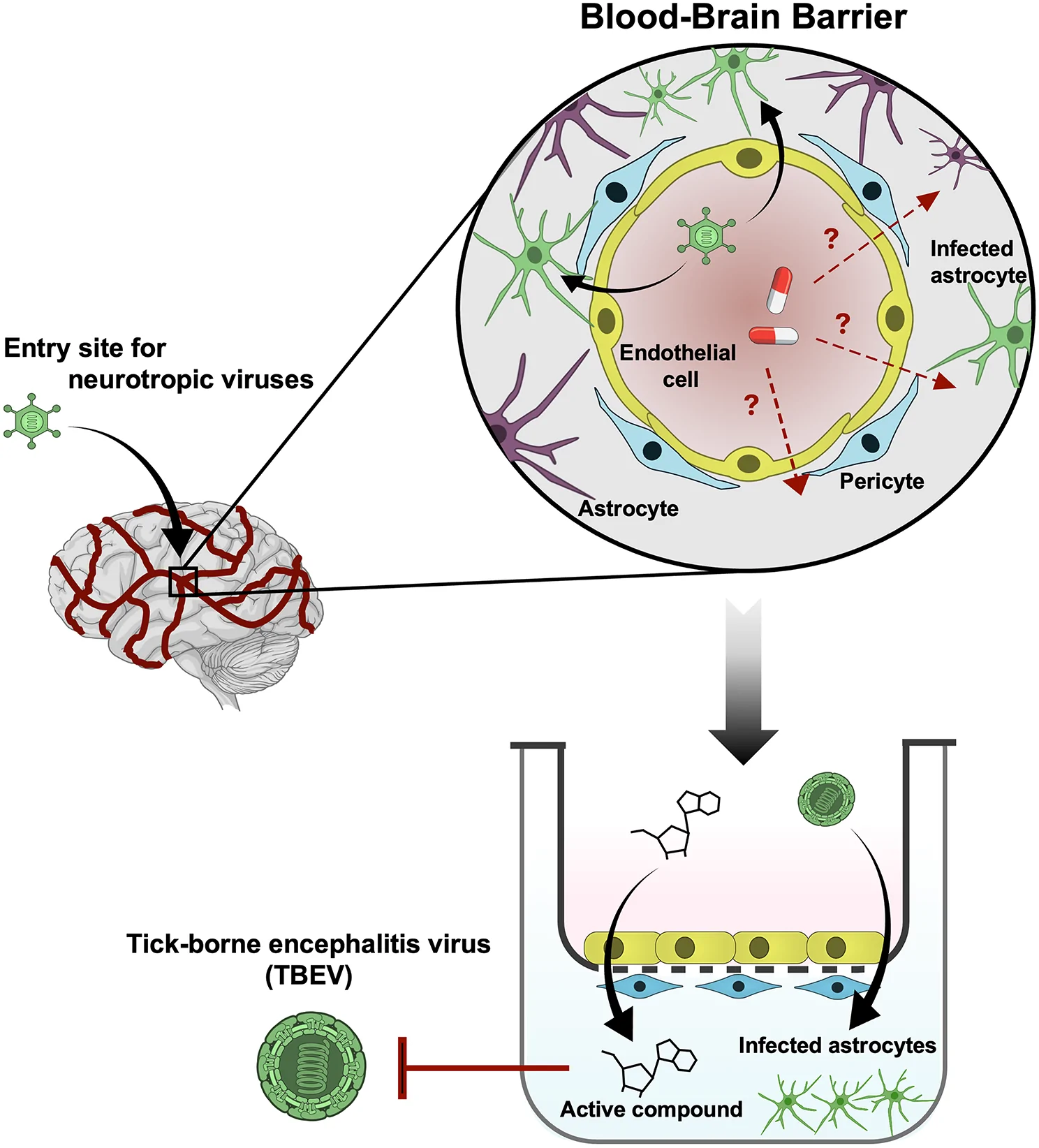
Modelling the human BBB for antiviral drug discovery and the study of viral infections

In this study, we established a cell culture model for the human blood-brain barrier (BBB) that was adapted to assay the passage of antiviral drugs and to study BBB infection of neurotropic viruses (TBEV) (Fig. 7). The core unit of the in vitro human transwell BBB model consisted of primary human brain microvascular endothelial cells (HBMEC) and pericytes (HBMVP). We observed the co-culture of these cells significantly increased barrier tightness in comparison to monocultures (Fig. 2E). Other in vitro studies have shown that brain endothelial cell and pericyte co-culture is sufficient to establish a tight barrier [68–70].

For drugs and neurotropic viruses, like TBEV, a critical access point into the CNS is the BBB. The BBB is composed of brain endothelial cells, pericytes, and astrocytes that together promote a tight and highly selective barrier. We established an in vitro BBB transwell model consisting of human brain microvascular endothelial cells and pericytes. The application of the model in an antiviral screening assay (BASA) resulted in the discovery of a novel BBB-permeable antiviral active against TBEV. Through the addition of human astrocyte-enriched cultures, we could investigate the passage and infection of TBEV at the BBB. The following images were incorporated into the schematic: NIAID Visual & Medical Arts. (10/7/2024). Brain Lateral. NIAID NIH BIOART Source.bioart.niaid.nih.gov/bioart/60; and NIAID Visual & Medical Arts. (3/20/2025). Zika Virus. NIAID NIH BIOART Source. bioart.niaid.nih.gov/bioart/604.

Antiviral drug discovery is faced with many challenges beginning from the design and synthesis of compounds to their efficacy in clinical trials [71]. It is frequently observed that the success of a drug in in vitro and in vivo models does not always translate in clinical trials. This discrepancy is often due to the limited availability of models that recapitulate human viral infection. For neurotropic viruses and CNS-targeting drugs, the BBB is a key access point [11, 23]. In vitro modelling of the BBB has long been valued in drug discovery for CNS disorders as it offers useful predictive information on the uptake potential of drugs [72]. With the wide availability of BBB models, not a single but the application of various models during drug development are proposed to help expedite the development of CNS therapeutics [73]. Building on this knowledge, our screening assay (BASA) offers a system to help identify BBB-permeable compounds by the functional assessment of a compound’s antiviral activity (Fig. 1).

The set-up of our BBB in a transwell model provides selection flexibility of viral cell targets for drug screening assays, potentially making BASA expandable to multiple neurotropic viruses (Fig. 1). This is an important feature since neurotropic viruses have distinct tropisms for neural cells. For example, HIV is described to infect astrocytes and microglia [74, 75]. In vitro data suggest that TBEV infects astrocytes, neurons, and oligodendrocytes [61–63]. The validation of BASA with anti-HIV drugs revealed there is a low-level passage of SAQ and T20, drugs with low CPE scores (Fig. 3D and E). For both compounds their CPE scores were changed from 0 to 1 for the 2008 to 2010 scores, respectively [47]. This reflects the difficulty in assigning the impermeable property of a compound in vivo. However, the ability to distinguish between these compounds and anti-HIV drugs with high CPE scores reveals there is selectivity in BASA (Fig. 3). This selectivity that agrees with a clinically derived ranking system, CPE scores. Overall, BASA is a tool that may help in identifying BBB-permeable antivirals and bridge the gap between pre-clinical and clinical efficacy of antiviral drugs targeted towards neurotropic viruses.

To provide further insight to TBEV interaction with the BBB, we characterized TBEV infection in our BBB model. In agreement with the literature, we observed low TBEV infection in HBMEC (Fig. 4A and D) [52, 76]. The low HBMEC infectivity is not unique to TBEV as it also described for Zika virus (ZIKV, *Flaviviridae*), Japanese Encephalitis Virus (JEV, *Flaviviridae*), and Rift Valley Fever Virus (RVFV, *Bunyaviridae*) [78–81]. Low infectivity of brain pericytes is also described for TBEV [77], and RVFV [78]. In our study TBEV antigen was not detected in HBMVP (Fig. 4B). This may be a result of differences in experimental conditions or pericyte heterogeneity [79]. For example, distinct responses to inflammatory stimuli, like lipopolysaccharide (LPS), were reported between two populations of human brain pericytes distinguished by the presence or absence of CD90 expression [39]. The HBMVP in our model are NG2 positive and we observed inconsistent expression of α-SMA and PDGFR-β, other pericyte markers (data not shown). In Prančlová et al., the TBEV-infected pericytes stained positively for PDGFR-β [77]. The differences in cell marker expression may indicate distinct pericyte populations that could result in diverse susceptibilities to TBEV infection. A recent study has shown that pericytes may restrict the passage of neurotropic viruses in a human model of the BBB [80]. These data highlight the importance in deciphering the role of pericytes during viral infections and their contributions to pathogenesis in future studies.

Astrocytes are not only important in supporting the BBB, but they are key cellular players during viral infections of the CNS [20, 55]. Here we demonstrate that human astrocytes derived from an immortalized human neural stem cell line, HNSC.100, are susceptible to TBEV infection (Fig. 4C and D). Astrocytes functioning as susceptible targets to TBEV infection is further supported by studies utilizing primary and human neural progenitor cell-derived astrocytes [61, 63, 81]. Like in these studies, we demonstrate that HNSC.100-differentiated astrocytes allow for productive TBEV infection (Supplementary Fig. 10, No BBB). In response to TBEV infection human astrocytes are also described to mount a robust inflammatory response [61, 62, 82]. For future investigations, characterizing the TBEV-induced responses in HNSC.100-differentiated astrocytes are needed to not only further validate these cells for TBEV infection models, but to determine their contribution to the neuroinflammatory environment.

We further demonstrate that our BBB model can be combined with astrocyte-enriched cultures to study TBEV infection in a tri-culture unit of the BBB. We observed TBEV crosses our in vitro BBB model to significantly infect HNSC.100-differentiated astrocytes without compromising barrier integrity (Fig. 5). Neurotropic *Flaviviruses*, including TBEV are suggested to use various non-exclusive mechanisms at the BBB to enter the brain [83]. These mechanisms include transcellular passage through endothelial cells, infected immune cells (Trojan horse), and paracellular passage (BBB breakdown) [83]. In the absence of BBB disruption in our model suggests that TBEV may utilize transcellular mechanisms to cross the BBB. In support of our observations, studies with HBMEC monocultures reported that TBEV crosses the BBB via transcellular pathways without compromising barrier integrity [52, 76]. In addition, in vivo mouse models indicate that TBEV entry to the CNS does not require BBB breakdown [84]. These data along with our observations support the use of transcellular entry mechanisms by TBEV. However, a combination of BBB passage mechanisms may be utilized during human infection that enhance TBEV dissemination in the brain.

In our tri-culture BBB model we also observed a delay of TBEV infection in HNSC.100-differentiated astrocytes (Fig. 5). This delay could implicate a few possibilities with one being the involvement of TBEV replicating in endothelial cells for transmission to astrocytes. The second could be the induction of host inflammatory responses that influence TBEV infection kinetics. The expression of inflammatory genes, like chemokines, in pericytes and astrocytes are described during TBEV infection [61, 62, 77, 82]. In addition, the products of interferon-stimulated genes (ISGs), viperin and TRIM79α, were reported to inhibit TBEV replication [85, 86]. These types of host responses may hinder TBEV replication in HBMEC and HBMVP but may also influence the infection kinetics in astrocytes. Through understanding the mechanisms employed in BBB passage and the TBEV-induced host responses, future studies could provide insight into viral passage and control at the BBB to unveil novel therapeutic targets.

Nucleoside analogues are small molecules that target viral polymerases, and they have long been valued in antiviral development [87, 88]. Along with their cost-effective synthesis, another advantageous quality of nucleoside analogues is that many have broad-spectrum activity against multiple viruses [89]. One such compound is ribavirin (RBV), first discovered in 1972, it showed activity against several RNA and DNA viruses with an unclear mechanism of action (MoA) [90]. RBV is approved for Hepatitis C virus (HCV) and reported to be active against TBEV [65, 91]. Initially, compound 3d, another nucleoside analog, was reported to exert antiviral effects against Zika virus (ZIKV), another neurotropic *Orthoflavivirus* [29]. With the application of BASA, we describe for the first time that compound 3d crosses the BBB and retains its antiviral activity against TBEV (Fig. 6C and E). Unlike compound 3d, RBV did not readily cross the BBB (Fig. 6B and D). These penetration efficiency differences may be a result of efflux transporters expressed on brain endothelial cells, like those belonging to the ATP-binding cassette (ABC) gene family [19, 92]. ABC transporters are described to influence drug distribution in the brain since a broad range of drug classes including antiretrovirals are substrates [19, 26]. As a result, there is an interest in inhibitors against ABC transporters like P-glycoprotein (Pgp) to improve the success of drugs in treating CNS diseases [19, 93]. This further highlights the importance of establishing in vitro BBB screening models to identify antivirals that can successfully cross the BBB without extensive hindrance of efflux transporters.

In this study we developed an experimental model for the BBB and adapted it to the identification of compounds that deliver antiviral activity through the BBB. We provide data encouraging the application of BASA as a tool to screen for BBB-permeable antivirals. With the application of TBEV and human astrocytes, we further demonstrate that our BBB (HBMEC and HBMVP) model can be adapted to investigate virus transmission through an intact BBB. However, the application of this model to studying mechanisms of BBB disruption and neuroinflammatory events has not been addressed yet. The inclusion of immune cells, exogenous inflammatory factors, and molecular profiling of neuroinflammatory markers would be required for future investigations on the interplay of neuroinflammation and viral infection at the BBB. The continued exploration of the potential of our multicellular BBB model may help to expand our understanding of viral and host mechanisms employed during infection and their potential role in CNS pathogenesis.

## Conclusions

Clinical drug development is an extensive and expensive process faced with a 90% failure rate of drug candidates [94]. This inherent limitation represents a significant obstacle in antiviral drug discovery where financial support is limited, and research efforts were historically deprioritized until the COVID-19 pandemic. Antiviral drug discovery for neurotropic viruses faces another challenge, the restricted access to the CNS. Our BBB model in combination with antiviral screening assays (BASA), provides a pre-clinical tool that could increase the success rate of antiviral drug candidates against neurotropic viruses. This is provided by the functional information offered by our model, which not only tests whether the compound crosses the BBB, but if it remains active to exert antiviral effects.

Along with the physical hindrance posed by the BBB, a general problem in CNS drug discovery is the shortage of appropriate animal models that effectively translate to human clinical studies [95]. The availability of a multicellular experimental model for the human BBB enables early testing of antiviral drug candidates before animal experimentation. Thus, the application of our model can help reduce the use of animal testing by providing a bottleneck for antiviral drugs with greater odds of targeting neurotropic viruses. This in turn helps promote the three R principle of animal research: Replacement, Reduction, and Refinement [96].

The BBB is not only a key access point for the delivery of antiviral drugs, but also for neurotropic viruses. The combination of human astrocytes with our BBB model provides a system to investigate the mechanisms utilized by neurotropic viruses to cross the BBB, which remain unclear. In addition, characterizing the host responses induced in co-culture models may provide insight into how BBB cell crosstalk influences infection and viral pathogenesis. By understanding viral infection at the BBB, there is opportunity to harness those mechanisms to not only prevent further CNS infection but deter the onset of neurological complications.

## Supplementary Information

Below is the link to the electronic supplementary material.

Supplementary Material 1

## Data Availability

The datasets used and/or analysed during the current study are available from the corresponding author on reasonable request.

## Abbreviations

ABC: Abacavir
BASA: BBB-permeable antiviral screening assay
BBB: Blood-brain barrier
CC_50_: 50% of maximal cytotoxicity concentration
CD31: Cluster of differentiation 31
CNS: Central nervous system
CPE: CNS penetration effectiveness
CSF: Cerebral spinal fluid
DPI: Days post-infection
FITC: Fluorescein isothiocyanate
HBMEC: Human brain microvascular endothelial cells
HBMVP: Human brain microvascular pericytes
HIV: Human immunodeficiency virus
HNSC.100: human neural stem cell line.100
HPI: Hours post-infection
IC_50_: Half maximal inhibitory concentration
IC_90_: 90% of maximal inhibitory concentration
LogP: Log of the partition coefficient
MOI: Multiplicity of infection
NEV: Nevirapine
NG2: Neural/glial antigen 2
RBV: Ribavirin
RPLP0: Ribosomal protein lateral stalk subunit P0
SAQ: Saquinavir
SI: Selectivity index
SN: Supernatant
TBEV: Tick-borne encephalitis virus
TEER: Transendothelial electrical resistance
TNF-α: Tumor necrosis factor-α
T20: Enfuvirtide
VEH: Vehicle
3d: Compound 3d
