# Supplementary material for: Establishment of an in vitro human blood-brain barrier model for antiviral screening against neurotropic viruses
Source: Fluids Barriers CNS. 2026 Sep 24;23:111. doi: 10.1186/s12987-026-00860-z (PMC13602755; doi:10.1186/s12987-026-00860-z)
Supplement: Supplementary file 1 [file 12987_2026_860_MOESM1_ESM.docx]

Supplementary Material

Establishment of an *in vitro* human blood-brain barrier model for antiviral screening against neurotropic viruses

Marisol Zuniga^1^, Alexander Herrmann^1^, Matteo Rizzato^1^, Michal Himl^5^, Adriana Fontes^2^, Hans Zischka^2,3^, Romano Silvestri^4^, Andrea Brancale^5^, Joachim J Bugert^6*^ and Ruth Brack-Werner^1,7*^

^(1-6)^ Affiliations of the authors are given in the main manuscript

**Supplementary Figure 1: Growth curves of primary HBMEC at different seeding densities.**

The ability of cultures generated with different seeding densities of primary HBMEC to achieve cellular confluence was monitored with the xCELLigence RTCA technology that measures cellular impedance. Values are expressed as cell index (CI). Maximum confluence corresponds to the plateau of CI over time. Data presented as the mean ± SD (n = 3). Statistical significance was determined by Ordinary one-way ANOVA with Tukey’s multiple comparisons test. *****p* ≤ 0.0001. Non-significant differences were not shown.

**Supplementary Figure 2: Co-culture of primary HBMEC and HBMVP significantly reduces the passage of FITC-conjugated dextrans with different molecular weights.**

(A-C) After 48 hours of incubation, barrier integrity of transwell inserts with HBMEC monocultures (HBMEC) or HBMEC and HBMVP co-culture (HBMEC+HBMVP) was determined by measuring the leakage of FITC-dextran. Inserts were incubated with 1 mg/mL of dextrans for 1 hour with the following molecular weights: (A) 4 kilodalton (kDa), (B) 10 kDa, and (C) 40 kDa. Data are expressed as fold change relative to the fluorescence values of HBMEC monocultures (HBMEC). Data presented as the mean ± SD (n = 5). Statistical significance was determined by Paired t-test. ***p* ≤ 0.01, *****p* ≤ 0.0001.

**Supplementary Figure 3:** **Titration of antiretrovirals in LC5-RIC to determine IC_50_.**

(A-D) Serial dilutions of nevirapine (NEV, A), abacavir (ABC, B), saquinavir (SAQ, C), and enfuvirtide (T20, D) were simultaneously added with HIV-1 inoculum (MOI 0.5) to LC5-RIC. Changes in HIV infection were fluorescently measured after 72 hours. Infection is expressed as percentages relative to fluorescence values of DMSO-treated LC5-RIC inoculated with HIV-1. Non-linear regression curves and IC_50_ were calculated using a sigmoidal dose-response with variable slope equation (red line). Data presented as the mean ± SD (n = 3).

**Supplementary Figure 4: Effects of antiretrovirals on primary HBMEC and HBMVP viability.**

(A, B) Primary HBMEC (A) and HBMVP (B) were incubated with serial dilutions of nevirapine (NEV), saquinavir (SAQ), abacavir (ABC), and enfuvirtide (T20). After 24 hours, cell viability was fluorometrically measured using cell titer blue reagent (Promega). Data is shown as percentages relative to fluorescence values of HBMEC or HBMVP treated with vehicle only (VEH). Data presented as the mean ± SD (n = 3). Statistical significance was determined by Kruskal-Wallis test with Dunn’s multiple comparisons test. **p* ≤0.05. Non-significant differences were not shown.

**Supplementary Figure 5: Antiretroviral inhibitory concentrations in the basal supernatants from BASA.**

(A-D) Drug concentrations in the basal supernatants (SN) from nevirapine (NEV, A), abacavir (ABC, B), saquinavir (SAQ, C), or enfuvirtide (T20, D)-exposed BBB (HBMEC+HBMVP) or No BBB (no cells) inserts. Each antiretroviral was titrated in parallel of BASA and a non-linear regression model applied to the dose-response curve. Drug concentrations were then calculated by the application of these curves to the antiviral effects reported in Figure 3. The predicted drug concentrations in No BBB basal SN would be equivalent to the drugs’ IC_90_ (dotted line). Data presented as the mean ± SD (n = 3). Statistical significance was determined by Unpaired t-test. ***p* ≤ 0.01. Non-significant differences were not shown.

**Supplementary Figure 6: Higher magnification images of TBEV-infected HBMEC and HNSC.100-differentiated astrocyte monocultures at 3-days post-infection (dpi).**

Immunofluorescence images of HBMEC and HNSC.100-differentated astrocyte monocultures infected with different multiplicity of infection (MOI) of TBEV Hypr at 3-dpi. Cells were stained with ⍺-*Flavivirus* envelope protein (E, red) and Hoechst 33342 (nuclei, blue). Images were acquired with a 20x objective.

**Supplementary Figure 7: TBEV does not disrupt primary HBMEC monolayer integrity.**

(A, B) HBMEC monolayer integrity was monitored with the xCELLigence RTCA technology that measures changes in cellular impedance, which are expressed as cell index (CI). (A) TBEV Hypr was added at different multiplicity of infection (MOI) to HBMEC monolayers (0 h) and monitored for 48 h. (B) Serial dilutions of TNF-α were added to HBMEC monolayers (0 h) and monitored for 48 h. Data is expressed as normalized cell index relative to the last cell index value before stimuli addition. Data presented as the mean ± SD (n = 4). Statistical significance was determined by Ordinary one-way ANOVA with Dunnett’s multiple comparisons test. **p* ≤0.05, ****p* ≤ 0.001, *****p* ≤ 0.0001. Non-significant differences were not shown.

**Supplementary Figure 8: Differentiation of HNSC.100 results in enriched population of GFAP^+^ and AQP4^+^ cells.**

**(**A) Progenitor cell line, HNSC.100, is cultured for 14 days in media lacking growth factors, EGF and FGF-2. By day 14, the differentiation results in an enriched population of astrocytic-like cells. (B) Transcript levels of astrocytic markers, glial fibrillary acidic protein (*GFAP*), aquaporin 4 (*AQP4*), and S100β (*S100B*) on day 0 (D0) and day 14 (D14) of differentiation. Transcript levels are expressed relative to housekeeping gene, *RPLP0*. (C) Immunofluorescence images of GFAP (green) and AQP4 (red) staining of D0 and D14 differentiated HNSC.100. Nuclei were stained with Hoechst 33342 (blue). Data presented as the mean ± SD (n = 3). Statistical significance was determined by Mann-Whitney test. ***p* ≤0.01.

**Supplementary Figure 9: TBEV does not induce significant death in monocultures of HNSC.100-differentiated astrocytes.**

(A, B) HNSC.100-differentiated astrocytes were inoculated with TBEV Hypr at different multiplicity of infection (MOI) and harvested at 1-, 2-, and 3-days post-infection (dpi) for flow cytometry. (A) The percentage of dead cells was monitored with Viakrome 808 fixable viability dye (Beckman Coulter). (B) Changes in glial fibrillary acidic protein (GFAP) expression were measured using a PE α-GFAP antibody (BD Biosciences). Data presented as the mean ± SD (n = 3). Non-significant differences were not shown.

**Supplementary Figure 10: TBEV infectious titers in basal supernatants from HNSC.100-differentiated astrocytes cultured with BBB (HBMEC+HBMVP) or No BBB inserts.**

Infectious viral titers in the basal supernatants (SN) from TBEV infection experiments presented in Figure 5 were determined by the median tissue culture infectious dose 50 (TCID50) assay. A549 cells were incubated with 10-fold serial dilutions of basal SN. Cytopathic effects (CPE) were visualized with a crystal violet solution. The Reed-Muench method was utilized to calculate the virus titer (TCID_50_/mL). Data presented as the mean ± SD (n = 3). Statistical significance was determined by Ordinary one-way ANOVA with Tukey’s multiple comparisons test. **p* ≤0.05, ***p* ≤0.01.

**Supplementary Figure 11: Titration of compound 3d and ribavirin in U251 MG.**

(A, B) Two-fold serial dilutions of compound 3d (3d, A) and ribavirin (RBV, B) were simultaneously added with TBEV Hypr inoculum (MOI 0.1) to U251 MG. After 72 hours, effects on TBEV infection were detected by immunofluorescence staining of the *Flavivirus* envelope (E) protein and images along with the fluorescence signal were acquired with the Incucyte SX-5 (Sartorius). Representative overlay images of bright field and E protein staining (red) are shown. Infection is expressed as percentages relative to fluorescence values of DMSO-treated U251 MG inoculated with TBEV. (C) U251 MG were incubated with two-fold serial dilutions of 3d and RBV for 24 hours. Cell viability was fluorometrically measured using cell titer blue reagent (Promega). Data is shown as percentages relative to fluorescence values of media-only treated cells. Non-linear regression curves and IC_50_/CC_50_ were calculated using a sigmoidal dose-response with variable slope equation (red line). Data presented as the mean ± SD (n = 3).

**Supplementary Figure 12:** **Application of BASA shows Compound 3d is a BBB-permeable antiviral against Zika virus.**

(A, B) Application of BASA with compound 3d (3d) and Zika virus (ZIKV; Uganda strain obtained from the Bundeswehr Institute of Microbiology). Stage 1: 3d added to the apical side of HBMEC+HBMVP (BBB) and empty (No BBB) inserts. The same 3d concentration used for TBEV (Figure 6) was applied. Stage 2: After 24 hours (h), U251 MG cells were incubated with basal supernatants and ZIKV inoculum (MOI 0.1) for 72 h and ZIKV infection determined by the presence of viral antigen and RNA. (A) Cytometric detection of U251 MG cells positive for *Flavivirus* envelope (E) protein at 72-hours post-infection (hpi). Infection levels are expressed relative to E protein positive cells incubated with TBEV and basal supernatants from vehicle (VEH)-exposed BBB or No BBB inserts. (B) ZIKV RNA levels in U251 MG cells at 72-hpi. Levels shown relative to the housekeeping gene, *RPLP0*. Non-significant differences were not shown.

**Supplementary Table 1:** Molecular weight (MW) and LogP values of compounds tested in the BBB-permeable antiviral screening assay (BASA). All the log values of the partition coefficient (logP), except for compound 3d, were obtained from the National Center for Biotechnology Information, PubChem. RBV (CID 37542), SAQ (CID 441243), NEV (CID 4463), T20 (CID 16130199), and ABC (CID 441300).

**Supplementary Table 2:** Antibodies utilized for immunofluorescence and flow cytometry.

**Supplementary Table 3:** Primers used for RT-qPCR.
